# Megaprosthesis Total Knee Replacement Following Resection of Extensive Osteoblastic Osteosarcoma in the Distal Femur: A Case Report

**DOI:** 10.7759/cureus.56971

**Published:** 2024-03-26

**Authors:** Oussama El Alaoui, Ousama Jelti, Adnane Lachkar, Abdeljaouad Najib, Hicham Yacoubi

**Affiliations:** 1 Orthopedics and Traumatology, Faculty of Medicine and Pharmacy of Oujda, Mohammed First University of Oujda – CHU Mohammed VI Oujda, Oujda, MAR; 2 Orthopedics, Traumatology and Orthopedic, Mohammed VI University Hospital, Faculty of Medicine and Pharmacy, Mohammed First University, Oujda, MAR; 3 Orthopedic Trauma, Mohammed VI University Hospital, Faculty of Medicine and Pharmacy of Oujda, Mohammed First University of Oujda, Oujda, MAR; 4 Traumatology, Mohammed VI University Hospital, Faculty of Medicine and Pharmacy, Mohammed First University, Oujda, MAR

**Keywords:** bone cancer surgery, chemotherapy response, primary total knee arthroplasty, limb-salvage, osteosarcoma research

## Abstract

Osteosarcoma is the most common type of primary bone cancer, which usually appears in the distal femur. The diagnosis of this condition typically involves advanced imaging and tissue biopsy, as well as taking into account characteristic clinical and radiographic indicators. The treatment approach for distal femoral osteosarcoma is multidisciplinary and involves initial chemotherapy, followed by limb-sparing surgery, reconstruction of bone and soft tissue, and subsequent adjuvant chemotherapy. We present a case study of a 25-year-old male admitted with a blastic lesion in the distal femur, confirmed via open biopsy to be osteoblastic osteosarcoma. Further evaluation revealed multiple pulmonary nodular lesions, managed with chemotherapy. After four months, regression of the lesion was observed. Due to malignant clinical and imaging features, excision of the lesion and subsequent reconstruction were performed, utilizing a custom-made total knee arthroplasty. The excision encompassed the removal of the distal 14 cm of the femur, with histological examination confirming central osteoblastic osteosarcoma. Satisfactory outcomes were observed during a one-year follow-up, indicating promising results. Vigilance is crucial, especially in young patients with surface-type bone tumors, as this neoplasm requires consideration.

## Introduction

Osteosarcoma is a primary bone cancer commonly found in long bones' metaphyseal region. The distal femur, proximal tibia, and proximal humerus are the most affected locations [1]. Amputation was considered standard practice previously, upon diagnosis of the tumor. However, with the introduction of chemotherapy and a multidisciplinary approach in specialized centers, the prognosis has significantly improved [2].

It is now possible to reconstruct a segment of removed bone due to a malignant tumor with metal prostheses that mimic the size and joint of the excised segment. This provides an additional viable option [3].

In this report, we provide a detailed procedure for distal femur resection with reconstruction using a massive total knee prosthesis. The aim is to highlight the benefits of conservative treatment, combined with a properly tailored adjuvant approach.

## Case presentation

A 25-year-old man, with no past medical history, presented with a swelling on the lower part of his left thigh. He complained of persistent pain that worsened at night. The swelling continued to increase in size along with a concurrent increase in volume. The patient's general condition was marked by anorexia and asthenia.

**Figure 1:**
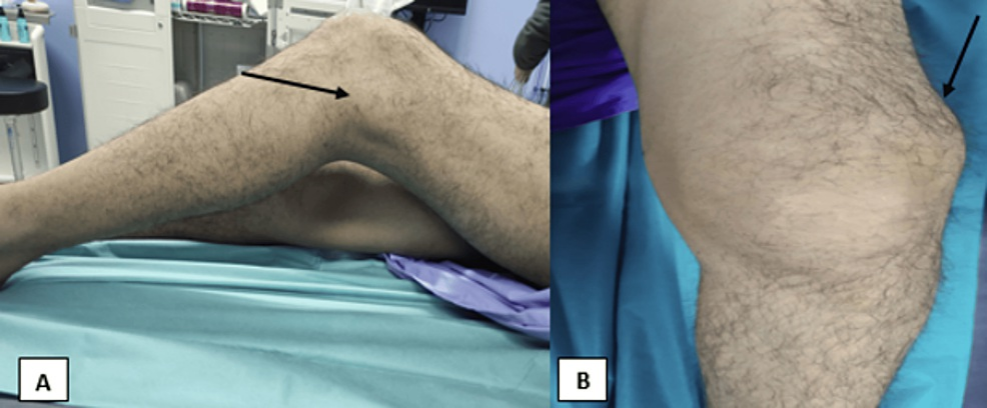
Clinical examination of the left knee at presentation. (A) Swelling of the lower section of the left tight with limitation of flexion of the knee. (B) The frontal aspect of the swelling on the anterolateral aspect of the lower section of the left thigh.

During the clinical examination, a swelling was observed on the front and side of the lower section of the left thigh. Reactive flushing was noted but there were no signs of inflammation. The patient's knee extension was limited to 40 degrees, while flexion was restricted to 100 degrees (Figures 1A, 1B).

**Figure 2:**
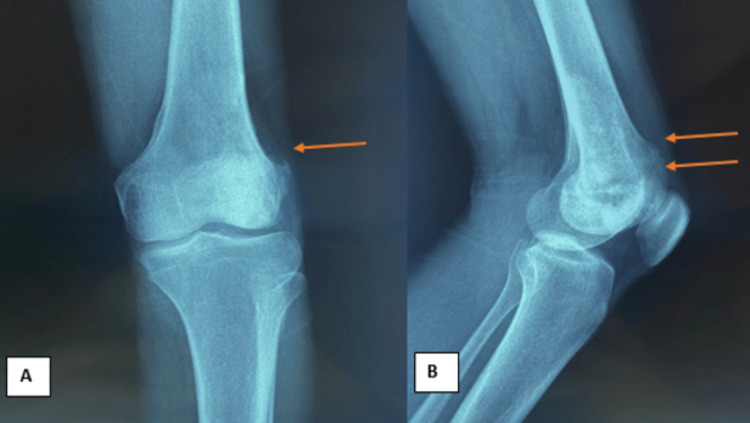
X-rays of the left knee during the initial presentation. (A) AP view reveals an osteoblastic epiphyseal-metaphyseal lesion. (B) Lateral view reveals the extension of the osteoblastic lesion to the entire external condyle.

The initial x-ray and CT scan revealed a blastic lesion located in the epiphyseal-metaphyseal region of the lower femur (Figures 2A, 2B).

Further examination revealed multiple pulmonary nodules, but no signs of any other secondary involvement. Biopsy confirmed the presence of osteoblastic osteosarcoma. The multidisciplinary team decided to start with neoadjuvant chemotherapy, followed by a resection-reconstruction surgery using a massive total knee prosthesis.

**Figure 3:**
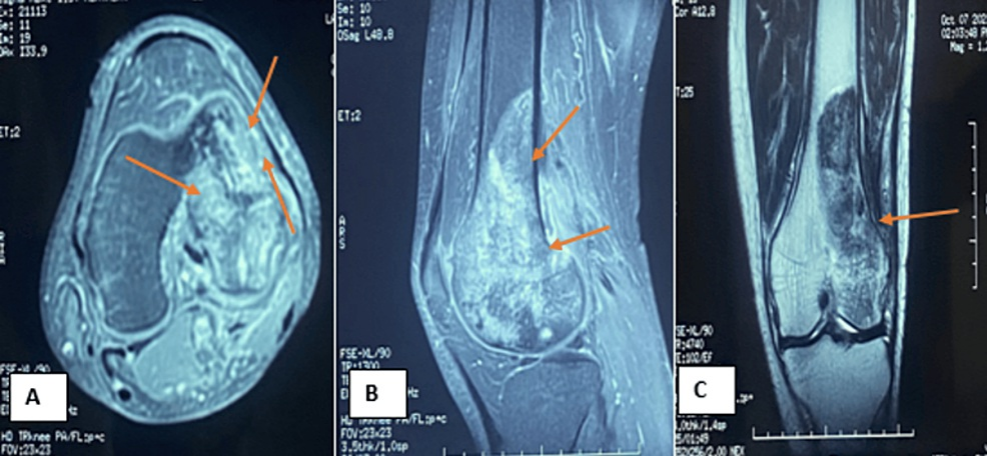
MRI of the patient after chemotherapy. (A) MRI coronal T2-Weighted image showing a osteoblastic lesion of the lateral condyle. (B) MRI sagittal T2-weighted image reveals the extension of the tumor to the femoral biceps muscle. (C) MRI axial T1-weighted image reveals the dimensions of the tumor.

After completing chemotherapy, the patient underwent an MRI of the knee. The MRI revealed a reduction in the metaphyseal-epiphyseal tumor process, which was characterized by T1 and T2 hyposignal. The affected region included the entire femoral condyle and the subchondral bone. The tumor also extended into adjacent compartments, notably affecting the biceps femoris muscle (Figures 3A-3C).

Under general anesthesia, the patient underwent surgical intervention, beginning with tumor removal. The extent of tumor resection was meticulously measured, maintaining a margin of 14 cm from the joint space. The excised tumor mass was then preserved in formalin for subsequent pathological analysis.

**Figure 4:**
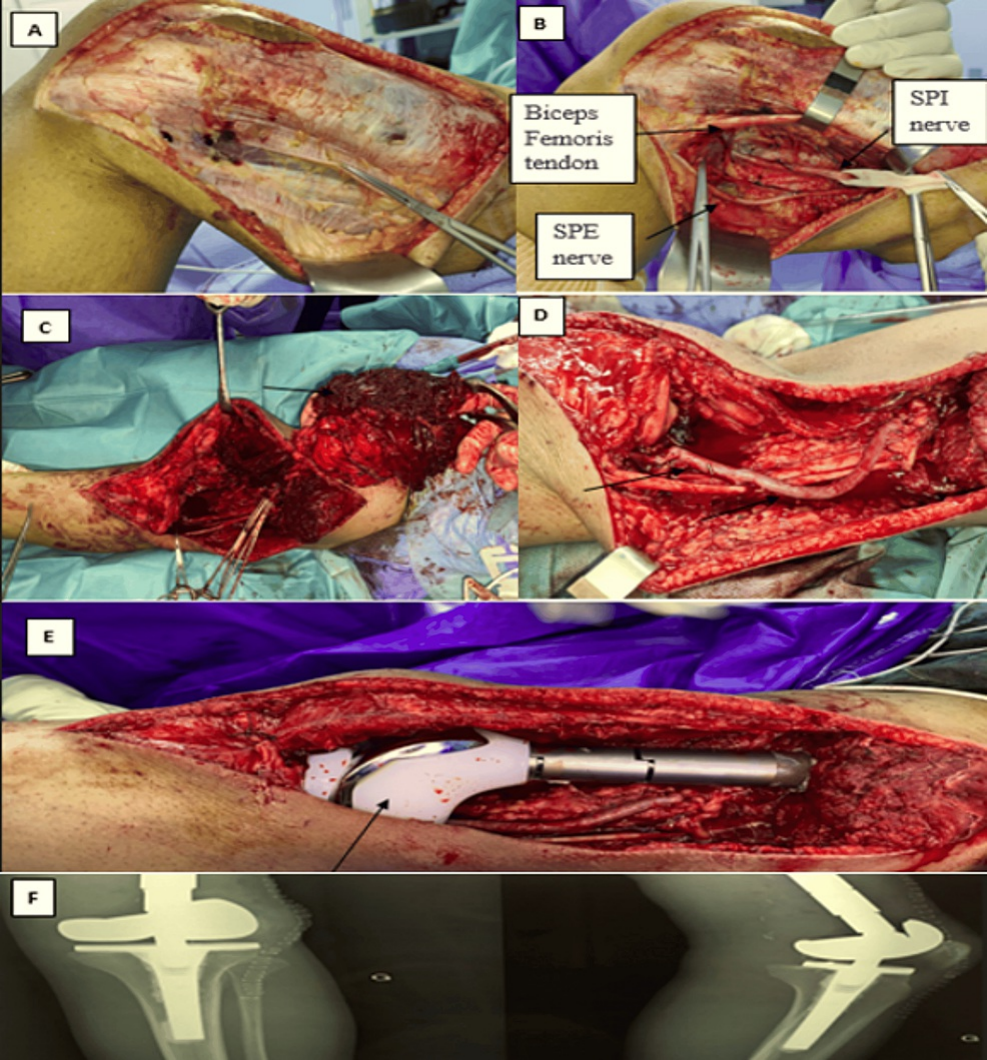
Intraoperative images of resection – reconstruction of the tumor. (A) Cutaneous and subcutaneous incision, centered on the tumor. (B) Neurolysis of the SPI and SPE nerves along with the lateral sural nerve. (C) Resection of the tumor. (D) Femoropopliteal bypass and ligation of the popliteal vein. (E) Placement of a definitive cemented massive knee prosthesis. (F) Postoperative x-rays.

During the second stage of the surgery, a femoropopliteal bypass was performed, and the popliteal vein was ligated after harvesting the saphenous vein from the patient's right lower limb. In the third stage of the surgery, the patient received a large left knee prosthesis, followed by a stability test (Figures 4A-4F).

**Figure 5:**
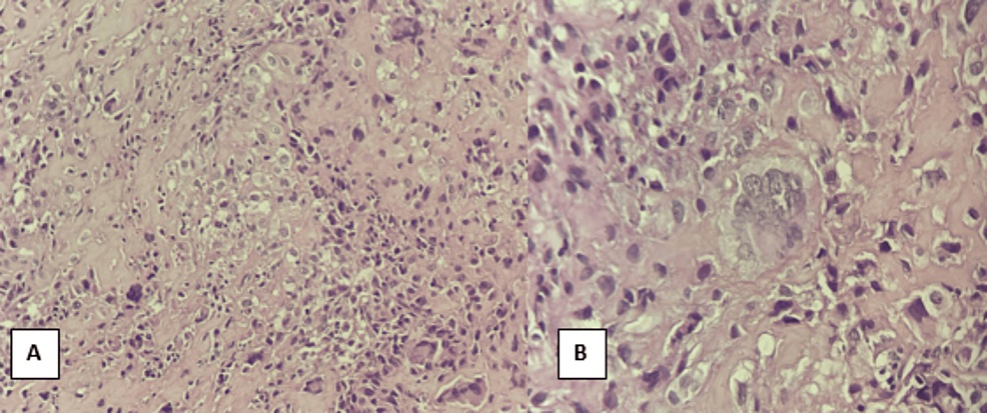
Microphotographs of the lesions at the anatomopathological examination (hematoxylin and eosin (H&E) Stain). (A) The lesion exhibits an oval-shaped morphology (magnification x 100). (B) Numerous atypical mitotic figures and extensive tumor necrosis (magnification x 40).

The results of the anatomopathological examination indicate the presence of a malignant sarcomatous growth that has spread out in diffuse layers, forming trabeculae and islands. The tumour was composed mainly of calcified osteoid. The tumor cells have an oval or spindle-shaped morphology, with abundant cytoplasm and enlarged nuclei exhibiting pronounced anisokaryosis. Numerous atypical mitotic figures are evident, along with extensive areas of tumor necrosis, confirming the diagnosis of osteoblastic osteosarcoma (Figures 5A, 5B).

After undergoing adjuvant chemotherapy using the same protocol, pulmonary nodular lesions were stabilized over the course of a year, and the patient experienced satisfactory functional outcomes.

## Discussion

Osteosarcoma is more common in adolescents and tends to start in areas where the bone is growing rapidly. Osteosarcoma is a highly aggressive cancer that needs immediate attention and treatment [1]. 25% of diagnosed patients show distant metastases, which can be detected through radiological imaging [4].

Radiology is very important in diagnosing suspected cases of osteosarcoma. It helps in evaluating the nature and extent of the local disease and detects any regional or distant metastases. Osteosarcomas can appear as purely osteolytic or osteoblastic, but they usually have a mixed appearance on radiographs [5]. MRI of the femur or thigh is acquired to assess the extent of marrow disease, identify and evaluate a potential soft-tissue component, detect skip metastases, and determine the involvement of critical neurovascular structures [6].

Limb salvage has become the standard of care in most cases of extremity sarcoma [3]. When there are large segmental bony defects, the primary methods of reconstruction usually involve either metallic implants (endoprostheses) or bulk allografts [7]. Endoprostheses have certain benefits over allografts, such as lower disease transmission risk and the possibility of early mobilization. However, they cannot restore bone stock or provide anatomic locations for soft tissue attachments [8].

In this context, modular rotating-hinge endoprostheses have become the preferred standard of care due to enhanced performance. However, the issue of stem fixation remains a subject of controversy [9]. Modular rotating-hinge distal femoral endoprostheses fixed with cement have proven to have a long-term survival rate of about 77%. Infections, tumor recurrence or mechanical issues are common early causes of failure, whereas implant fatigue or aseptic loosening are attributed to late failures [10,11].

In the initial period after surgery, it is important to concentrate on regaining movement, especially bending and straightening. According to a study by Schwartz et al, patients who received cemented modular distal femoral endoprostheses achieved an average of 110° flexion but had a mean extensor lag of 6° [12].

In our case, distal femoral reconstruction was accomplished using a cemented modular expandable knee endoprosthesis, resulting in positive functional outcomes. The procedure involved the removal of the distal femur and accompanying soft tissue mass, intending to achieve sufficient margins in both bone and tissue. The patient's response to chemotherapy appears to have contributed to a favorable outcome and stabilization of the metastatic lesion.

## Conclusions

The management of osteosarcoma that affects the distal femur requires a multidisciplinary approach. The success of conservative surgical treatment relies heavily on the administration of appropriate and effective neoadjuvant chemotherapy. Our clinical case emphasizes the importance of prosthetic replacement in the surgical intervention for osteosarcoma of the distal femur, even when there is metastasis. This highlights the developing role of prosthetic replacement as a vital component in the comprehensive treatment strategy for this challenging malignancy.
